# Food Consumption Characteristics and Influencing Factors in a Grassland Transect of Inner Mongolia Based on the Emergy Method

**DOI:** 10.3390/foods11223637

**Published:** 2022-11-14

**Authors:** Mengmeng Jia, Lin Zhen

**Affiliations:** 1 Institute of Geographic Science and Natural Resources Research, Chinese Academy of Sciences, Beijing 100101, China; 2 University of Chinese Academy of Sciences, Beijing 100049, China

**Keywords:** food consumption, emergy method, agro-pastoral zones, Inner Mongolia

## Abstract

Food consumption is the vital connection between human beings and natural resources. Our research explores the characteristics and drivers of food consumption patterns within Mongolian grasslands with a vulnerable ecology and environment. Food consumption data were obtained via a household questionnaire survey, and the emergy method was applied to analyze the food consumption characteristics in different grassland areas. This led to the following results: (1) The total per capita food consumption in different banners showed greater consumption and higher percentage of animal-based food in regions farther north. (2) From south to north, the main meat consumption in different banners varied, the predominant meat type consumed in Taibus was pork, that in Zhenglan was mutton, and that in West Ujimqin was beef. The farther north, the more fruits and vegetables were consumed. (3) The characteristics of food consumption in different areas were influenced by a series of factors, including social, economic, and ecological ones. Local food supply and disposable income were the main factors that had important effects on food consumption structure, as these two factors provided accessibility to foods for consumption.

## 1. Introduction

Food is the basic and solid foundation for human survival and plays a key role linking human beings and nature. Food consumption structure reflects not only development in the economy, but also in the local livelihoods and diets of residents. Sustainable and healthy food consumption is vulnerable to a series of factors, especially external causes such as rapid population growth, worldwide environment pollution, global food crisis, and ecological degradation. These challenges can hinder food consumption and security [1,2,3,4].

The income of households and individuals rises with the development of economy and society, and this leads to an upward trend in food consumption diversity [5]. Some researchers have found that food consumption can give expression to the consumption, utilization, and occupation of ecosystem services by human production and life [6], and there are mutual influences between human lifestyle and food consumption behaviors [7,8,9]. For instance, unsustainable food consumption will inevitably cause great pressure on local ecosystems and restrict regional development [10]. 

In order to explore the change in food consumption, prior research has mainly focused on food consumption structure [11,12,13], food consumption behavior [7,9,14,15,16], influencing factors [17,18], food nutrients [19,20,21], and the influence of foods on the environment [14,22,23,24,25]. During the research process of food consumption, main methods such as the ecological footprint, water footprint, carbon footprint, and AIDS mode have been used widely [26,27,28,29]. The data in prior studies mainly depended on statistical data and lacked unique evaluation standards for food consumption, which limited the depth and effectiveness of the studies. 

Adopting scientific and feasible methods contributes to study food consumption change, which achieves some performance. Previous research accumulated some foundation on the evolution of food consumption, food production and its influencing factors in different levels [11,12,30,31], apart from the specific content of food consumption, the regular and popular methods contain ecological footprint evaluation [27,29,32,33], modeling [34,35,36], and household interviews [11,37,38]. Many scholars have developed some meaningful research methods to study or evaluate the regular pattern of temporal or spatial variations of food consumption and production. However, these popular methods still have some insufficiencies. For instance, it is hard for them to overcome the unique evaluation standard among different systems such as ecology, economy, and society. Emergy analysis satisfies this unique measurement standard and can be used to compare and analyze varied factors on different levels. 

Emergy can be widely applied in complex research systems by measuring elements as energy obtained from the sun. Emergy can be used to describe specific products or services, and solar energy Joules (seJ) is typically used as the measurement unit for emergy [39,40]. As emergy is originally used to express that the biosphere obtains energy from the sun directly or indirectly, emergy analysis provides a unified unit of measurement for different products and services in varied systems [41,42,43]. As to specific product research on wheat, maize and milk, the emergy method typically has been used to analyze the efficiency of production [43,44,45,46]. When it comes to the study of a complicated system such as agriculture, emergy analysis can make valid links and better define relationships between the economy, environment, and land use [47,48,49,50]. 

Xilingol grassland, as the main part of the Xilingol League, is located in the east part of Inner Mongolia, and it is one of the four major grasslands in China. The ecological environment is one of constant vulnerability. Studying the characteristics of food consumption in Xilingol grassland is of great significance to the study of food consumption characteristics of residents in grassland areas in China. Based on questionnaire survey data from face-to-face interviews with farmers and herdsmen in the Xilingol League in 2019, the Xilingol Economic and Social Statistical Yearbook, and the national land use data from 2015, this paper mainly aims to (1) explore the quantity and structure of farmers’ and herdsmen’s food consumption in a Xilingol grassland transect, (2) compare food consumption characteristics among the typical areas of the grassland transect, and (3) delineate factors influencing variation in food consumption characteristics. Survey data results can serve to benchmark residents’ food consumption to help formulate food policy changes in the Xilingol League.

## 2. Methods

### 2.1. Study Area 

**Figure 1:**
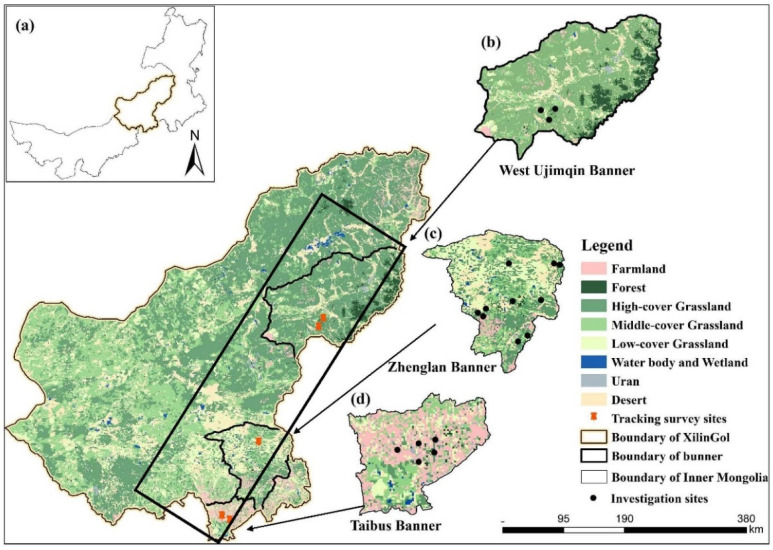
Location of the study area. (**a**) Inner Mongolia, (**b**) West Ujimqin Banner, (**c**) Zhenglan Banner, (**d**) Taibus Banner. Data resources: Location data were obtained from the Global Positioning System (GPS) when we took household interview and land use data, which were obtained from the Resource and Environment Science and Data Center (http://www.resdc.cn, accessed on 2 August 2022).

The Xilingol League is an extremely important part of China’s Inner Mongolia Autonomous Region, which is located in the central part of this region at 42°32′ to 46°41′ N and 111°59′ to 120°00′ E (Figure 1). It is a part of the Mongolian Plateau with elevation ranging from 800 to 1800 m above sea level. In this league, annual precipitation averages 288 mm with an annual pan evaporation of 1700 to 2600 mm. In 2020, the annual average temperature of the whole League was 3.8 °C, which was 0.4 °C lower than that in 2019. The annual average precipitation of the League in 2020 was 326 mm, which is 18% higher than that of a normal year and 17% higher than that of 2019. 

In 2020, the registered population of the Xilingol was 1,040,000 people. Among them, the urban population was 476,000, while the agricultural and pastoral areas totaled 565,000 residents. The urbanization rate of household registration was 45.77%. There were 45 ethnic groups in the League, including 729,000 Han, 335,000 Mongolians, and 291,000 Manchu. The Xilingol League covers an area of 200,000 km^2^. This includes 180,000 km^2^ of grassland and 5860 km^2^ of forest, making up 90% and 2.9% of the area, respectively. In 2020, the sown area of grain crops was 144,000 hectares (ha), which was an increase of 0.3% compared to 2019. Annual grain output in 2020 was 465 million kilograms (kg), an increase of 3.3% compared to the previous year. By the end of 2020, the per capita disposable income of residents was Chinese Yuan (CNY) 33,495 (USD 4750.07) per year, which was an increase of 3.2% compared to that in 2019. The per capita disposable income of urban residents was CNY 41,391 (USD 5869.84) per year, an increase of 1.5% over that in 2019, versus CNY 18,864 (USD 2675.19) per year for rural residents, which was 8.5% more than the previous year. CNY to USD conversions were made on 21 September 2022 (https://www.xe.com/currencyconverter/convert/).

Xilingol Grassland is mainly distributed in the Xilingol League. In this study, three banners with typical areas in the Xilingol Grassland were selected for investigation and survey. From north to south, the three areas hold different land use characteristics: the West Ujimqin Banner belongs to a typical pastoral grassland area whose main production mode is grazing; part of the Hunshandake Sandy Land distributes in the Zhenglan Banner, which has a main production mode of grazing and supplemented production mode of farming; different from the two banners ahead, the main production mode in Taibus Banner is farming, as it belongs to the agro-pastoral transitional zone (Figure 1). The annual precipitation in these three banners shows differences to some extent. In the northernmost banner, West Ujimqin, its average annual precipitation is 350 mm, with a range of 189 to 564.5 mm. The annual precipitation in Taibus is similar to that in West Ujimqin. Compared with these two banners, the annual precipitation in Zhenglan banner is a little higher, at 365 mm. Precipitation in arid and semi-arid areas is insufficient, which contributes to their degradation.

### 2.2. Data Resources

We conducted a survey of how residents consume food in different geographical zones from south to north in the Xilingol League. By choosing and interviewing local typical households that lived in the rural areas of the three banners since 20 July in 2019, we obtained much meaningful information about the characteristics of their food consumption during more than two weeks. Data were obtained by asking respondents to fill out a questionnaire survey during household interviews. Altogether, 145 households were interviewed, including 47 from West Ujimqin Banner, 52 from Zhenglan Banner, and 46 from Taibus Banner. Surveyed households were randomly selected. The chosen principles were covering the main areas of the banner and selecting the representative households. For instance, West Ujimqin banner contains five towns and two villages; our surveyed areas covered three towns and two villages. Zhenglan banner mainly holds 3 towns and four villages; our survey areas covered two towns and two villages. Taibus banner contains five towns and one village, while our survey areas covered three counties. In general, we chose interviewees from family members who knew more about their family’s total food consumption and some other specific details such as the household size, income, education, and age. The food consumption categories of rural families in the Xilingol Grassland were mainly grains, meat, vegetables, fruits, egg, milk, and cereals [11]. In this survey, we did not design any normalized questionnaire to lead the interviewees to obtain the results we wanted. We helped interviewees to find the factors that they thought were important, instead of obtaining their feedback to prechosen factors that we deemed significant. We mainly applied open questions, such as: “what foods do you choose in daily life?” Basic demographic information on the interviewees was obtained, including their age, education, household size, and ethnic group (Table 1). When analyzing the influencing factors of food consumption change, data on GDP, disposable income, and population were obtained from the government website of the Inner Mongolia Autonomous Region (http://tj.nmg.gov.cn/ accessed on 5 August 2022) and the Xilingol League government (http://www.xlgl.gov.cn/zjxlgl/ accessed on 5 August 2022).

### 2.3. Methods 

As to energy flow, the energy quality is different in different periods. Emergy metric solar energy Joules (seJ) represent the beginning point to various products or services. When the emergy transformation relationship can be found, different products or services can be compared by using the emergy as the unique standard. 

Our research aimed to study changes in food consumption and its characteristics in typical areas of Inner Mongolia. The foods we need usually were obtained from the sun directly or indirectly. Different foods had different conversion relationships with the sun. The weight of food consumption can be achieved by survey questionnaire (Appendix B). The calculations involving the consumption of different foods mainly put all the food items into a unique standard using a conversion rate (Table 2). The equation expressing emergy comes from [28]. The specific and detailed calculation equations are as follows:

(1) $$C_{i}=G_{i}\times F_{i}\times R_{i}$$

(2) $$C_{P}=\sum_{i=1}^{m}C_{i}\;(m=1,\;2,\;3\ldots \ldots 6)\;$$

(3) $$C_{A}=\sum_{i=10}^{n}C_{i}\;(n=7,\;8,\;9\ldots \ldots 13)\;$$

where *i* is the food item number, from 1 to 13, $G_{i}$ is the gravity of food consumption, $F_{i}$ is the energy conversion rate, $R_{i}$ is emergy transformity, $C_{i}$ is the specific kind of food consumption emergy, $C_{P}$ is total plant-based food consumption emergy, and $C_{A}$ is total animal-based food consumption emergy.

## 3. Results 

### 3.1. Food Consumption Structure and Characteristics in Different Banners

**Figure 2:**
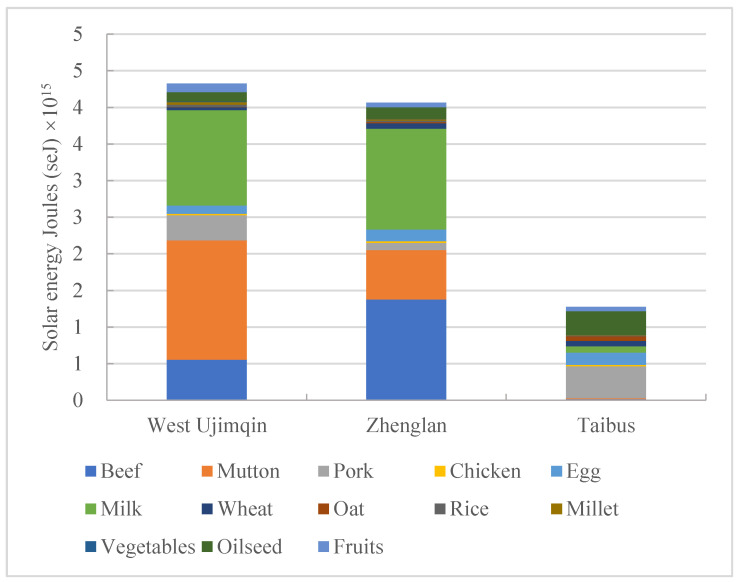
The structure of food consumption in the three banners.

Although the components of food consumption in the West Ujimqin, Zhenglan, and Taibus banners were similar, the total per capita annual food consumption and the specific dominant food categories were different among these three banners. In West Ujimqin banner, it was found that in all food consumption categories, the most consumed food was mutton, for which the consumption percentage was 37.78% of the total food consumption quantity (Figure 2). Per capita annual milk and beef consumption accounted for 1.30 × 10^15^ and 5.54 × 10^14^ seJ, and their percentages of total food consumption were 30.21% and 12.83%. The cumulative consumption percentage of these three kinds of foods exceeded 80%. Thus, food consumption in West Ujimqin banner is mainly focused on animal-based food, with little dependence on plant-based foods such as wheat and rice. 

When compared with food consumption in West Ujimqin, one of the dominant characteristics of food consumption in Zhenglan banner was that the percentage of beef consumption was the greatest, at 33.88% of the total food consumed. The per capita annual consumption percentages of milk and mutton were 33.87% and 16.59%, respectively. The cumulative consumption percentage of these three kinds of food was more than 83%. Plant-based food consumption of wheat and rice accounted for 7.39 × 10^13^ and 1.88 × 10^13^ seJ, with consumption percentages of 1.82% and 0.46%, respectively. Food consumption in Zhenglan banner mainly depends more on animal-based food than on plant-based food, similar to that in West Ujimqin banner. 

Food consumption in Taibus banner had one outstanding feature in that meat consumption mainly depended on pork, unlike West Ujimqin meat consumption focusing on mutton or Zhenglan meat consumption focusing on beef. In Taibus, pork consumption occupied 89.88% of total meat consumption. Apart from pork, the other two main kinds of food consumed were egg and oilseed, for which the consumption percentages out of the total food consumption were 13.40% and 25.55%, respectively; this differed from the other two banners’ secondary food consumption types, such as milk, beef, and mutton. Plant-based food consumption such as that of wheat and oats in Taibus accounted for 7.49 × 10^13^ and 6.46 × 10^13^ seJ, respectively, with consumption percentages of 5.90% and 5.09%, respectively, of the total food consumption. These values are much higher than those for the other two banners. When we compared the total food consumption in three banners, total food consumption in Taibus was much lower than the other two banners. This was due to the consumption of beef, mutton, and milk being much lower than the other two banners. Although residents in Taibus consume more grain than in the other two banners, the emergy transformity of grain is rather low compared to beef, mutton and milk.

### 3.2. Animal- and Plant-Based Food Consumption in the Three Banners

While comparing food consumption in these three banners, it was found from the geographical location analysis that the further south the region is, the greater the plant-based food consumption and the smaller the animal-based food consumption. Regarding the per capita annual total food consumption, West Ujimqin and Zhenglan had similar consumption quantities, accounting for 4.32 × 10^15^ and 4.07 × 10^15^ seJ, respectively. The Taibus banner’s total food consumption per capita accounted for only 1.27 × 10^15^ seJ, which was lower than that in West Ujimqin by 68.80% and that in Zhenglan by 70.60%.

**Figure 3:**
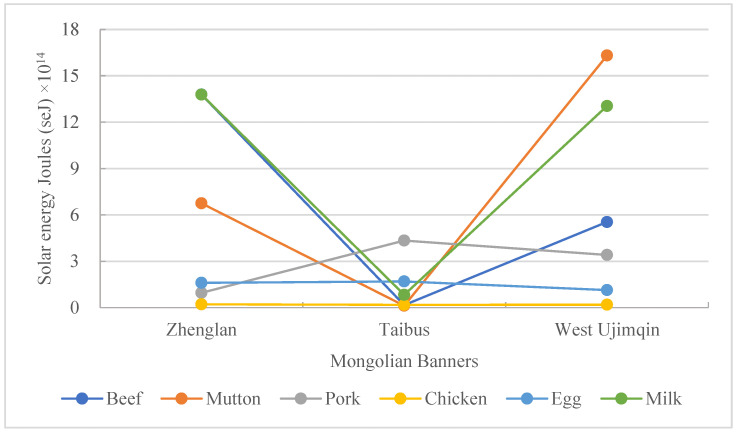
A comparison of animal-based food consumption in the three banners.

It was significant that in terms of animal-based food consumption, the value for Taibus was much lower than those for the other two banners (Figure 3). In particular, the most consumed meat in the different banners was also different: that in West Ujimqin was mutton, that in Zhenglan was beef, and that in Taibus was pork. The greatest chicken consumption appeared in Zhenglan, but the difference for this consumption type across the three banners was not large: the value for Zhenglan was more than that in West Ujimqin by 13.80% and that in Taibus by 16.39%. The egg consumption amounts in Zhenglan and Taibus were similar, accounting for an average quantity of 1.65 × 10^14^ seJ; this was more than that in West Ujimqin by 45.34%. Milk consumption showed a significant difference among the three banners: the greatest consumption appeared in Zhenglan and was much higher than that in Taibus—by 15.52-fold. 

**Figure 4:**
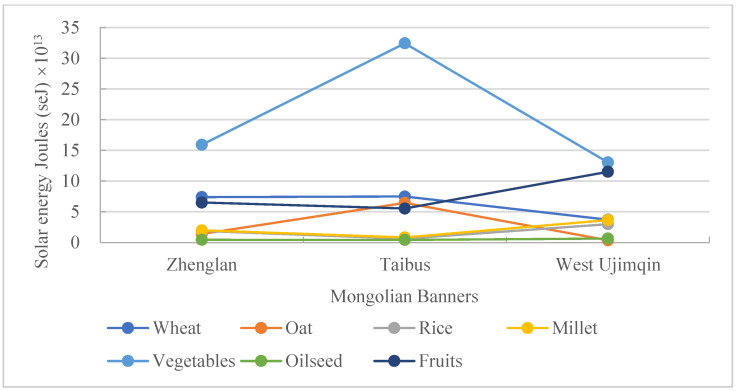
A comparison of plant-based food consumption in the three banners.

The characteristics of plant-based food consumption in the three banners were different from those of animal-based food consumption. From north to south, total consumption of plant-based foods increased further south. Plant-based food consumption in Taibus banner came to 5.38 × 10^14^ seJ, which was much higher than that in other two banners, by about 50% (Figure 4). However, there was only a small difference in plant-based food consumption between the other two banners—only 1.28%. As to specific plant-based food consumption, such as of rice, millet, vegetables, and fruits, the consumption in West Ujimqin banner was the greatest when compared with the other two banners. The greatest consumed quantities of rice, millet, vegetables, and fruits were 373.88%, 339.79%, 52.17%, and 108% higher, respectively, than those least consumed across the banners. The consumption of wheat, oats, and oilseed increased further south, with the greatest consumption of these three kinds of food appearing in Taibus banner, and the least appearing in West Ujimqin banner. The greatest consumed quantities of these three kinds of food were 1.01-fold, 16.43-fold, and 1.49-fold higher, respectively, than the least consumed quantities across the banners. 

### 3.3. Comparison of Animal- and Plant-Based Food Consumption Rates

**Figure 5:**
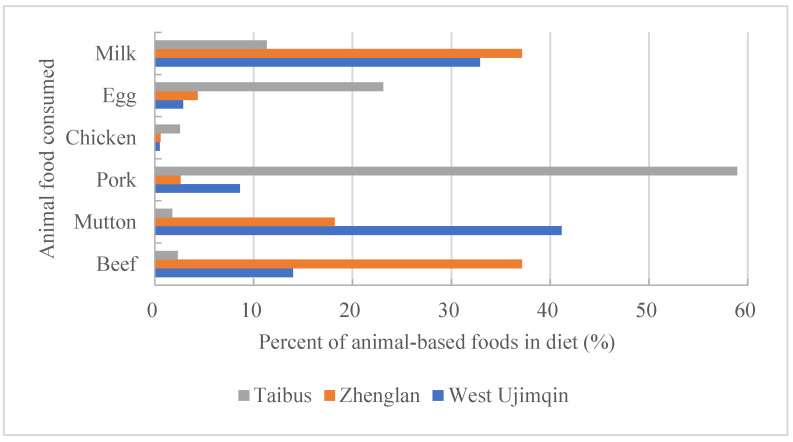
A comparison of animal-based food consumption ratios among the three banners.

Animal-based food consumption in these three banners was mainly represented by beef, mutton, pork, chicken, egg, and milk. When comparing the consumption rates of all animal-based foods across different banners, it was found that beef consumption in Zhenglan accounted for 37.16%, which was much higher than that in the other two banners (Figure 5). The mutton consumption rate in West Ujimqin was 41.16%; this was also the highest consumption rate for all the animal-based foods in this banner. This rate was much higher than that in other two banners. The highest consumption rate of pork was found in Taibus, coming to 58.93%. The chicken consumption rates in these three banners were all very low, with the highest being only 2.55%. The biggest egg consumption rate was 23.10% in Taibus, which was about 20 percentage points more than that in the other two banners. The milk consumption rate situation was different to that of egg. The highest consumption rate of milk was 37.14%, which happened in Zhenglan banner, and the lowest one was only 11.33%, in Taibus banner. 

**Figure 6:**
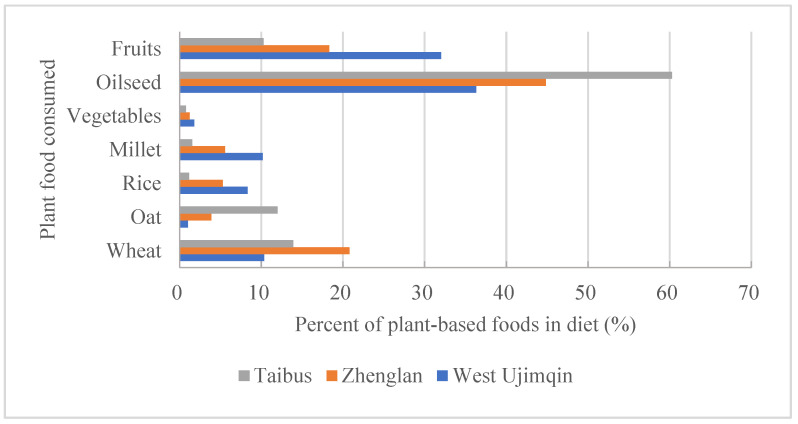
A comparison of plant-based food consumption ratios among the three banners.

Plant-based food consumption in these three banners was mainly represented by wheat, oats, rice, millet, oilseed, vegetables, and fruits. The most prominent proportion in Taibus was for oilseed, at 60.28% (Figure 6), which was much higher than that in the other two banners and was the highest contributor to the total plant-based food consumption. The highest consumption rate for oats appeared in the same banner as that for oilseed, but the highest consumption rate of oats was 12.01%, trailing far behind the rate for oilseed. The greatest consumption rates of rice, millet, vegetables, and fruits all appeared in West Ujimqin banner, and the least ones were found in Taibus banner. Different from other kinds of food consumption rates, the greatest rate for wheat, 20.82%, appeared in Zhenglan banner. The consumption rates of wheat in the other two banners were both more than 10%.

## 4. Discussion

### 4.1. Economic Factors

**Figure A1:**
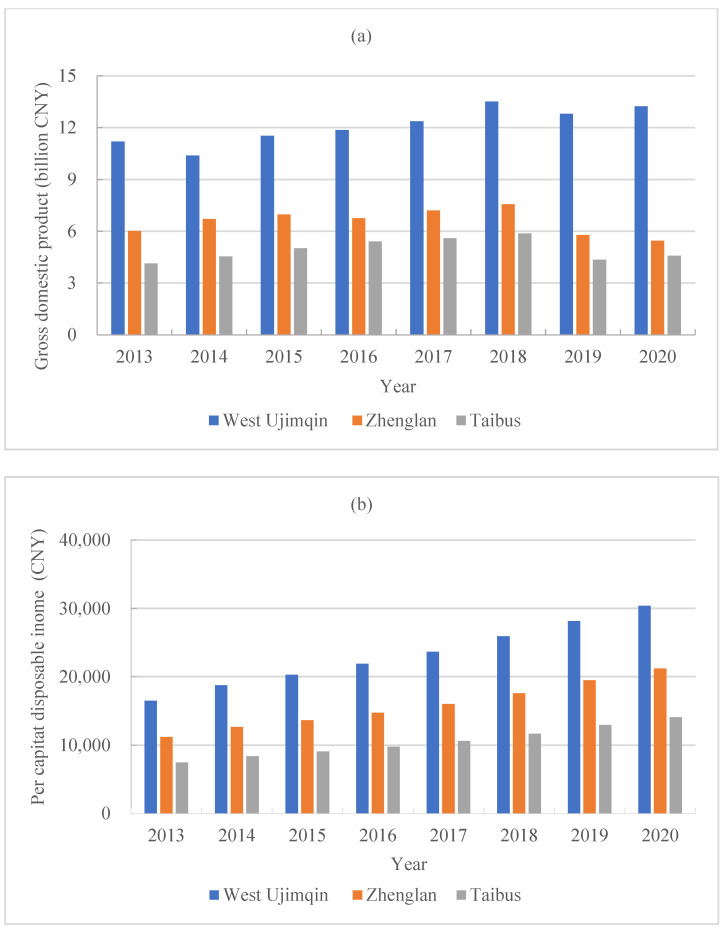
Gross domestic product (**a**) and per capita disposable income (**b**) of permanent residents of rural and pastoral areas in three Mongolia banners.

Gross domestic product (GDP) represents the value of final (versus intermediary) goods and services produced in a region or country over a set time such as a year. This factor has a significant role in improving residents’ disposable income and food consumption quantity, especially in rural and pastoral areas [13,51]. The GDP in these banners mainly increased, with West Ujimqin banner having the greatest GDP compared to the other two banners (Figure A1a). As important parts of the GDP, the net incomes from agriculture and animal husbandry in the three banners showed high growth rates of 13.3% and 14.7% compared with incomes in the previous year. Income growth provides a strong foundation for people to purchase more varied food [52]. The per capita disposable income of permanent residents of rural and pastoral areas also proves this point. The factor that plays a large role in food consumption quality is per capita disposable income, and related research has verified this result [53]. In these three banners, per capita disposable income was the highest in West Ujimqin (Figure A1b), where it was 47.38% higher than in Zhenglan banner and 122.19% higher than in Taibus banner. This mirrors West Ujimqin’s per capita annual food consumption being the highest, implying a relation between disposable income and food consumption characteristics (Table 3). 

### 4.2. Social Factors 

Population change reflects the total food consumption quantity and potential demand in the future [19]. In this study, the population of Taibus was much higher than that of the other two banners, by about 2.5-fold. The populations of the other two banners were similar. Although Taibus had a population advantage, its per capita food consumption was much lower than that of the other two banners, while the per capita disposable incomes of residents in this banner were lower compared to the other two banners. This hindered local residents’ ability to purchase food, which is why Taibus’ total food consumption was the lowest of the three banners. 

Besides local food supply, cultural differences in minority residents had an important effect on food consumption customs and preferences [54,55]. According to our survey results, the residents of West Ujimqin and Zhenglan belonged mainly to the Mongolian and Han ethnic groups. Mongolians accounted for more than 80% in West Ujimqin and more than 60% in Zhenglan. Meanwhile, the residents in Taibus were mainly of the Han ethnic group. Mongolians consumed much more meat and milk than Hans, while Han people consumed more plant-based foods such as grains and oilseed than Mongolians. Per capita consumption of fruits and vegetables in the West Ujimqin banner was much higher than in the other two banners, mostly because its population’s high disposable income allows them to purchase food items from the markets. Otherwise, age was also an influencing factor that cannot be neglected [56,57]. According to survey results, the age of the interviewed residents in Taibus banner were mainly over 50 years, as most younger adults went to cities for more income. Since local residents in West Ujimqin and Zhenglan banners had relatively higher income from grazing, more young people stayed in local villages. Since older people do not typically eat as much as young people, resident food consumption in Taibus banner was lower compared to the other two banners. 

### 4.3. Ecological Factors

The ecological environment is the basic and solid foundation for human life, providing many and varied foods [58,59]. Different food yields and livestock quantities guarantee typical food consumption. From north to south across these three banners, cultivated and sown area and yields of grain and oil-bearing crops were all greater. Those factors had a significant effect on residents’ food consumption [60,61]. For instance, the total sown area in Taibus was greater than in the other two banners (Figure A3b), which contributed to higher grain production in Taibus than that in the other two banners. The yields and per capita consumption of grain and oil-bearing crops in Taibus were greatest among the three banners studied (Figure A3a). 

**Figure A2:**
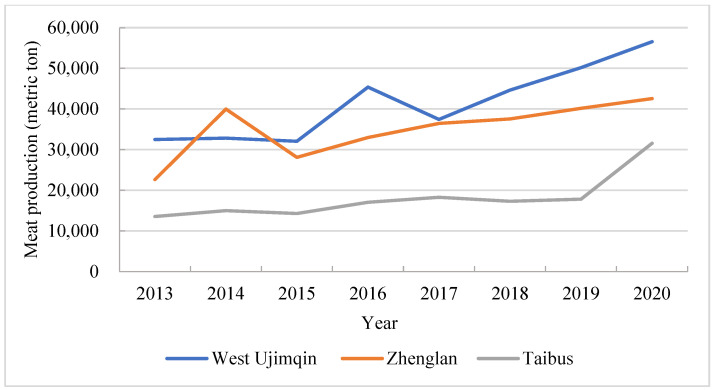
Annual production of meat in three Mongolian banners.

**Figure A3:**
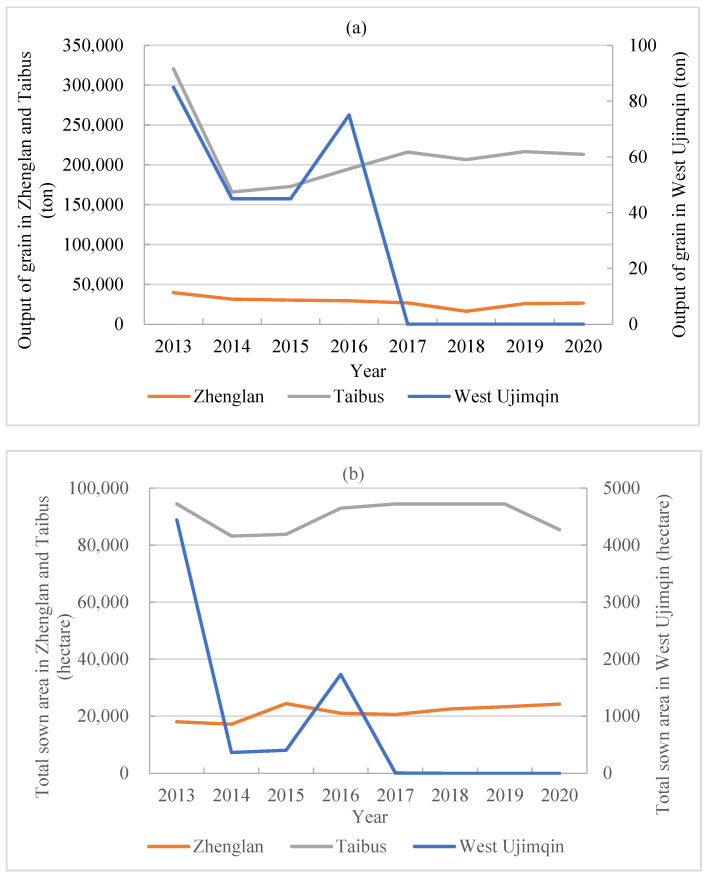
Total output of grain (**a**) and sown area (**b**) in three Mongolian banners.

As the total population increases, urbanization in these three banners also grows, which requires more food types and puts more pressure on land use [62]. Structural changes in food consumption results in more land use for production [18,63]. Increasing income stimulates the demand for meat production (Figure A2). Greater demand for meat can result in overgrazing and degradation of grassland environments [64,65]. Taibus banner historically has focused on crop production with recent cropland expansion due to increased demand (Figure A3b, Table 3). Increasing crop area combined with an aging population has had a negative effect on farmland use efficiency [66]. At the same time, consumption structure changes result in the adjustment of land use types [67]. It is well known that the natural environment and ecological conditions in this grassland transect are of fragile status and have become worse because of inappropriate land use [68,69]. In order to balance the local ecology with sustainable economic development, scientific management policy and diversified food supply methods are necessary. 

## 5. Conclusions

According to survey data obtained from three typical areas by personal interview, it was found that food consumption in different zones of the Xilingol grassland of the Inner Mongolia Autonomous Region showed significant spatial differences from north to south. The main results are as follows:

(1) The foods consumed represented plant-based and animal-based foods, mainly including beef, mutton, pork, milk, egg, grains, oilseed, and fruits. The total per capita food consumption in different banners showed greater consumption quantities and higher percentages of animal-based foods in regions further north.
(2) The main meat consumption in different banners showed obviously different characteristics from south to north. The predominant meats were pork in Taibus, mutton in Zhenglan, and beef in West Ujimqin. Regions further north showed higher fruit and vegetable consumption than other areas.
(3) The characteristics of food consumption in different areas were influenced by a series of factors, including social, economic, and ecological ones. Local food supply and income were the main factors that had important effects on food choice and consumption structure, as these two factors determine accessibility in food consumption [70,71].

Food consumption quality and quantity will improve with continuous economic development. Income and population increases contribute to more food production and more food categories. This inevitably results in more land being used for grain, vegetables and livestock [72]. This will put more pressure on natural resources and the fragile ecology of the region. 

Although this research showcased useful results and insights, there are some shortcomings. Because of the limited budget and time, it was difficult to survey more people. Expanding the scale of our survey in the future may bolster the accuracy and representativeness of our results. The emergy method can be expanded to other local food systems and regions with dietary contrasts of plant-based and animal-based foods. This can further enhance our understanding of the emergy content, tradeoffs, and impacts of food consumption choices.

## Figures and Tables

**Table 1:** Demographic information of survey respondents.

		West Ujimqin	Zhenglan	Taibus
Household size	One	0.00%	1.92%	8.70%
	Two	8.51%	13.46%	50.00%
	Three	38.30%	38.46%	17.39%
	Four	38.30%	32.69%	15.22%
	Five	6.38%	9.62%	4.35%
	Six	8.51%	3.85%	4.35%
Ethnic group	Mongolian	82.98%	61.54%	0.00%
	Han	17.02%	36.54%	100.00%
	Manchus	0.00%	1.92%	0.00%
Education	Illiterate	2.13%	3.85%	45.65%
	Under primary school	2.13%	3.85%	4.35%
	Primary school	34.04%	30.77%	21.74%
	Junior school	31.91%	34.62%	21.74%
	High school	23.40%	25.00%	6.52%
	University	6.38%	1.92%	0.00%
Age	30–40	44.68%	17.31%	0.00%
	41–50	38.30%	40.38%	10.87%
	51–60	17.02%	26.92%	19.57%
	61–70	0.00%	15.38%	58.70%
	71–80	0.00%	0.00%	10.87%

**Table 2:** Conversion rates of different food items [28].

Number	Item	Energy Conversion Rate (J/t)	Emergy Transformity (seJ/J)
1	Wheat	1.57 × 10^10^	6.80 × 10^4^
2	Oats	1.60 × 10^10^	8.00 × 10^4^
3	Rice	1.55 × 10^10^	3.95 × 10^4^
4	Millet	1.60 × 10^10^	8.00 × 10^4^
5	Oilseed	2.55 × 10^10^	6.90 × 10^5^
6	Vegetables	2.51 × 10^9^	2.70 × 10^4^
7	Fruits	3.30 × 10^9^	5.30 × 10^5^
8	Mutton	1.41 × 10^10^	2.00 × 10^6^
9	Pork	2.00 × 10^10^	1.70 × 10^6^
10	Beef	8.76 × 10^9^	3.17 × 10^6^
11	Chicken	5.40 × 10^9^	2.00 × 10^6^
12	Egg	8.30 × 10^9^	2.00 × 10^6^
13	Milk	2.90 × 10^9^	1.71 × 10^6^

**Table 3:** Correlations between per capita food consumption and several influencing factors. - represents that the data of factor in banner was too little to show correlation with per capita food consumption, ** represents significant at 1% level.

	Taibus Banner		Zhenglan Banner		West Ujimqin Banner	
Influencing Factors	Correlation Coefficient (*r*)	*p* Value	Correlation Coefficient (*r*)	*p* Value	Correlation Coefficient (*r*)	*p* Value
Per capita disposable income	0.593 **	<0.01	0.580 **	<0.01	0.766 **	<0.01
Meat production	-	-	0.511 **	<0.01	0.518 **	<0.01
Output of grain	0.475 **	0.01	-	-	-	-
Sown area	0.259	0.082	-	-	-	-

## Data Availability

Data is contained within the article.

## Appendix A

The figures in Appendix A show economic and agricultural characteristics of three Inner Mongolian banners. Figure A1 highlights gross domestic product and per capita disposable income. Figure A2 summarizes meat production. Figure A3 compares grain output and sown area.

## Appendix B

Appendix B shows the survey questionnaire used.
